# Synthetic Human TLR9-LRR11 Peptide Attenuates TLR9 Signaling by Binding to and thus Decreasing Internalization of CpG Oligodeoxynucleotides

**DOI:** 10.3390/ijms17020242

**Published:** 2016-02-22

**Authors:** Xichun Pan, Bin Li, Mei Kuang, Xin Liu, Yanyan Cen, Rongxin Qin, Guofu Ding, Jiang Zheng, Hong Zhou

**Affiliations:** 1 Department of Pharmacology, College of Pharmacy, the Third Military Medical University, Chongqing 400038, China; xichunpan@163.com (X.P.); libin6033@sina.com (B.L.); km0640602@126.com (M.K.); cenyanyan@163.com (Y.C.); michel_0415@163.com (R.Q.); dingguofu@126.com (G.D.); 2 Medical Research Center, Southwestern Hospital, the Third Military Medical University, Chongqing 400038, China; triplestars@163.com

**Keywords:** CpG oligodeoxynucleotides, TLR9, Toll-like receptor 9, synthetic peptide, proinflammatory cytokine, nuclear factor, phosphorylation, internalization

## Abstract

Toll-like receptor (TLR) 9 is an endosomal receptor recognizing bacterial DNA/CpG-containing oligodeoxynucleotides (CpG ODN). Blocking CpG ODN/TLR9 activity represents a strategy for therapeutic prevention of immune system overactivation. Herein, we report that a synthetic peptide (SP) representing the leucine-rich repeat 11 subdomain of the human TLR9 extracellular domain could attenuate CpG ODN/TLR9 activity in RAW264.7 cells by binding to CpG ODN and decreasing its internalization. Our results demonstrate that preincubation with SP specifically inhibited CpG ODN- but not lipopolysaccharide (LPS)- and lipopeptide (PAM3CSK4)-stimulated TNF-α and IL-6 release. Preincubation of SP with CpG ODN dose-dependently decreased TLR9-driven phosphorylation of IκBα and ERK and activation of NF-κB/p65. Moreover, SP dose-dependently decreased FAM-labeled CpG ODN internalization, whereas non-labeled CpG ODN reversed the inhibition. The K_D_ value of SP-CpG ODN binding was within the micromolar range. Our results demonstrated that SP was a specific inhibitor of CpG ODN/TLR9 activity via binding to CpG ODN, leading to reduced ODN internalization and decreased activation of subsequent pathways within cells. Thus, SP could be used as a potential CpG ODN antagonist to block TLR9 signaling.

## 1. Introduction

The innate immune system employs pathogen-recognition receptors (PRRs) such as Toll-like receptors (TLRs) and NOD-like receptors (NLRs) to sense pathogen-associated molecular patterns (PAMPs) [1]. TLR9 is a receptor that senses bacterial DNA/CpG-containing oligodeoxynucleotides (CpG ODN). The extracellular domain (ECD) of human TLR9 (hTLR9) comprises 25 leucine-rich repeats (LRR) that contribute to binding of CpG ODN [2,3]. Internalized CpG ODN within the endosome initiates TLR9-mediated signaling via sequential recruitment of MyD88, interleukin receptor associated kinase (IRAK) and TNF-α receptor associated factor 6 (TRAF6), which in turn activate important downstream transcription factors, such as NF-κB and AP-1, culminating in the induction of proinflammatory cytokines such as TNF-α and IL-6 [3].

TLR9 plays a crucial role in infectious diseases, autoimmune disorders and cancers [4]. Septic TLR9^−/−^ mice exhibited lower inflammatory cytokines release and higher survival compared with wild-type mice, indicating that inhibition of TLR9 is a potential therapeutic strategy for sepsis [5]. Additionally, in autoimmune diseases, such as rheumatoid arthritis and systemic lupus erythematosus, self-DNA/RNA-protein complexes also activate TLR9 [6]. Therefore, CpG ODN/TLR9 signaling could be considered a potential target for treating these diseases.

At present, a common strategy to block TLR9 signaling is to interfere with molecules involved in the pathway. MyD88-target inhibitors, such as BB-loop mimics, and TNF-α-target agents, such as adalimumab, have been used to inhibit TLR9 signaling [7]. Various TLRs and their adapter proteins have many universal molecules; therefore, MyD88-target inhibitors can destroy the immune system [8]. In addition, we investigated an inhibitory CpG ODN designed as a TLR9 antagonist to block immunostimulatory CpG ODN to treat sepsis and lupus nephritis [2]. However, its application was limited because of its low target-specificity [9]. Therefore, it is important to identify new inhibitors that target TLR9 or CpG ODN more specifically and safely.

Our previous report demonstrated that TLR9 with a deleted LRR11 had a lower response to CpG ODN than the wild-type TLR9 [10], and this mutated TLR9 had lost almost all its binding capacity for CpG ODN, suggesting that LRR11 is critical for binding CpG ODN. Therefore, in this study, a synthetic LRR11 peptide (SP) was synthesized, and its influence on TLR9 signaling was investigated.

## 2. Results and Discussion

### 2.1. SP Inhibits CpG ODN-Induced Cytokine Release from RAW264.7 Cells

#### 2.1.1. SP Decreases TNF-α and IL-6 Release Induced by CpG ODN from RAW264.7 Cells

CpG ODN activates MyD88-dependent TLR9 signaling, leading to the release of proinflammatory cytokines, including TNF-α and IL-6, which play crucial roles in inflammatory diseases and autoimmune disorders [11]. Therefore, the influence of SP on CpG ODN-induced TNF-α and IL-6 release from RAW264.7 cells was investigated. An unrelated peptide (URP) derived from LRR14 of human TLR5 was used as a control, according to our previous results [3].

**Figure 1:**
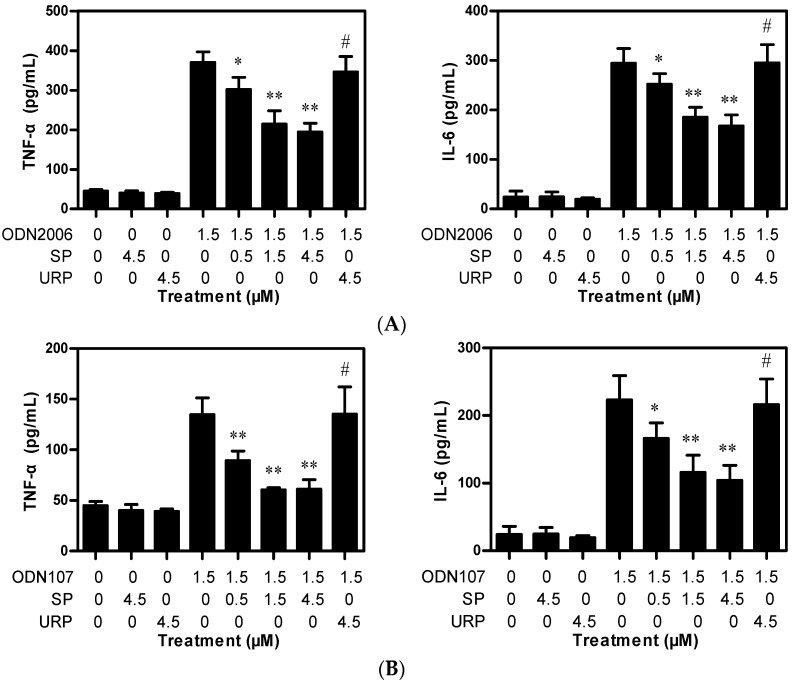
Synthetic peptide (SP) decreased TNF-α and IL-6 release from RAW264.7 cells treated with CpG oligodeoxynucleotides (CpG ODN). RAW264.7 cells (4.0 × 10^5^ cells) grown in 48-well plates were treated with 1.5 μM of ODN2006 or ODN107 preincubated with SP (0, 0.5, 1.5, or 4.5 μM) or unrelated peptide (URP) (4.5 μM) at 37 °C for 15 min. After incubation for 6 h, the supernatants were collected to detect TNF-α (**A**) and IL-6 (**B**) using enzyme-linked immunosorbent assay (ELISA) kits. Data from one of three independent experiments are shown (*n* = 4). “*”, *p* < 0.05 *vs.* ODN2006 or ODN107; “**”, *p* < 0.01 *vs.* ODN2006 or ODN107; “#”, *p* > 0.05 *vs.* ODN2006 or ODN107.

As expected, CpG ODN 2006 (hereafter referred to as ODN2006) or CpG ODN 107 (hereafter referred to as ODN107) significantly increased TNF-α and IL-6 release (Figure 1); however, SP or URP alone had no influence on cytokines release. Although lower concentration of SP (0.5 μM) had weak inhibitory effects on ODN2006-induced cytokines release, higher concentrations of SP (1.5 and 4.5 μM) showed a significant inhibitory effect. The inhibition ratios of SP were 41.9% (1.5 μM) and 47.4% (4.5 μM) for ODN2006-induced TNF-α release, and 36.9% (1.5 μM) and 42.9% (4.5 μM) for IL-6 release (Figure 1A). SP showed a similar effect on ODN107-induced cytokine release (Figure 1B). The inhibition ratios of SP were 55.1% (1.5 μM) and 54.6% (4.5 μM) for ODN107-induced TNF-α release, and 48.1% (1.5 μM) and 53.4% (4.5 μM) for IL-6. The results demonstrated that SP could decrease CpG ODN-induced TNF-α and IL-6 release from RAW264.7 cells dose-dependently.

#### 2.1.2. SP Inhibits CpG ODN-Induced, But Not LPS- and PAM3-Induced, Cytokine Release

**Figure 2:**
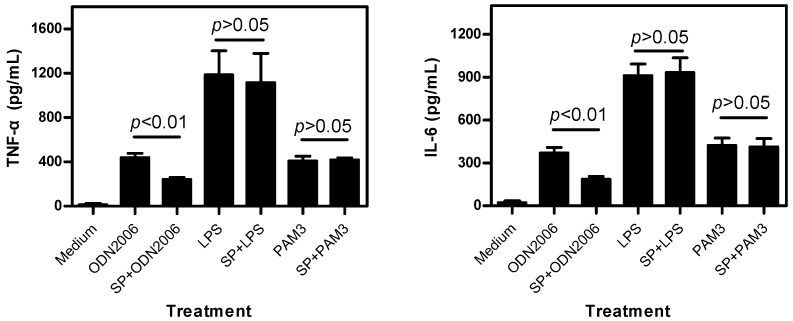
Synthetic peptide (SP) did not inhibit TNF-α and IL-6 release induced by lipopolysaccharide (LPS) or PAM3CSK4 (PAM3) from RAW264.7 cells. Experiments were performed as described in Figure 1. The concentrations of ODN2006, LPS, PAM3 and SP were 1.5 μM, 20 ng/mL, 5 μg/mL and 1.5 μM, respectively. Data from one of three independent experiments are shown (*n* = 3).

To detect the specificity of the inhibitory effect of SP on CpG ODN-induced cytokines release, the influence of SP on lipopolysaccharide (LPS, recognized by TLR4) or synthesized PAM3CSK4 (PAM3, recognized by TLR1/2)-induced TNF-α and IL-6 release was further investigated. As expected, ODN2006 (1.5 μM), LPS (20 ng/mL) and PAM3 (5 μg/mL) significantly increased the release of TNF-α and IL-6. Preincubation with SP (1.5 μM) dramatically inhibited cytokines release induced by ODN2006 (Figure 2). However, SP had no inhibitory effect on cytokines release induced by LPS or PAM3. The results demonstrated that SP specifically inhibited CpG ODN-induced, but not LPS- and PAM3-induced, cytokine release, suggesting that SP would affect only CpG ODN/TLR9-mediated, not LPS/TLR4 or PAM3/TLR1/TLR2-mediated, pathways.

### 2.2. SP Inhibits CpG ODN-Induced NF-κB Activation

**Figure 3:**
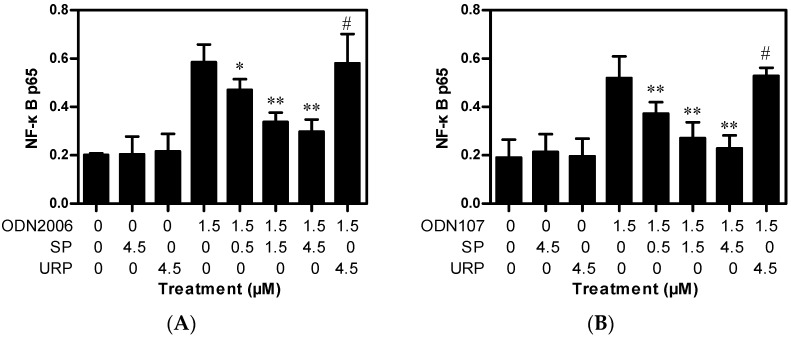
Synthetic peptide (SP) inhibited NF-κB activation induced by CpG oligodeoxynucleotides (CpG ODN) in RAW264.7 cells. RAW264.7 cells (5.0 × 10^6^ cells) grown in six-well plates were treated with 1.5 μM of ODN2006 (**A**) or ODN107 (**B**) preincubated with SP (0, 0.5, 1.5, or 4.5 μM) or unrelated peptide (URP) (4.5 μM) at 37 °C for 15 min. After incubation for 4 h, the cells were collected to extract nuclear proteins. The p65 subunit was detected using enzyme-linked immunosorbent assay (ELISA) kits. Data from one of three independent experiments are shown (*n* = 3). “*”, *p* < 0.05 *vs.* ODN2006 or ODN107; “**”, *p* < 0.01 *vs.* ODN2006 or ODN107; “#”, *p* > 0.05 *vs.* ODN2006 or ODN107.

CpG ODN/TLR9-mediated release of inflammatory cytokines depends on activation of nuclear factors such as NF-κB [10]. Therefore, the influence of SP on CpG ODN-induced NF-κB activation was investigated. The concentration of the p65 subunit of NF-κB in the total nuclear protein extract was detected using enzyme-linked immunosorbent assay (ELISA). The results showed that ODN2006 and ODN107 significantly increased the activation of p65 and that SP (4.5 μM) or URP (4.5 μM) alone had no effect. As expected, preincubation with SP (0.5–4.5 μM) significantly inhibited the NF-κB activation induced by ODN2006 or ODN107 in a dose-dependent manner (Figure 3). These results demonstrated that the inhibitory effect of SP on CpG ODN-induced cytokines release was associated with inhibition of NF-κB activation.

### 2.3. SP Inhibits CpG ODN-Induced IκBα and ERK Phosphorylation

**Figure 4:**
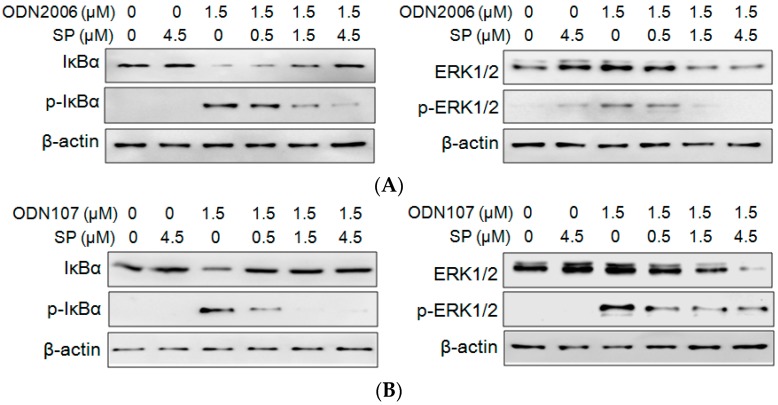
Synthetic peptide (SP) decreased the phosphorylation of IκBα and ERK1/2 in RAW264.7 cells treated with CpG oligodeoxynucleotides (CpG ODN). RAW264.7 cells (1.0 × 10^7^ cells) grown in 10-cm dishes were treated with 1.5 μM of ODN2006 (**A**) or ODN107 (**B**) pre-incubated with SP (0, 0.5, 1.5, or 4.5 μM) at 37 °C for 15 min. After incubation for 4 h, cells were harvested to extract total protein; and the protein expressions of IκBα, phosphorylated (p)-IκBα, ERK1/2 and p-ERK1/2 were detected by western blotting. Data from one of three independent experiments are shown.

CpG ODN/TLR9-mediated cytokines release depends on the phosphorylation of upstream kinases, such as inhibitory kappa B alpha (IκBα), and mitogen-activated protein kinases (MAPKs), such as extracellular regulated protein kinases 1/2 (ERK1/2) [12]. Therefore, the influence of SP on the phosphorylation of IκBα and ERK1/2 was investigated. The results showed that SP alone had no effect on the degradation and phosphorylation of IκBα (IκBα and p-IκBα). In cells treated with ODN2006, the levels of IκBα phosphorylation and degradation significantly increased. However, SP (0.5, 1.5 and 4.5 μM) decreased the degradation and phosphorylation of IκBα induced by ODN2006, and decreased the phosphorylation of ERK1/2 induced by ODN2006 (Figure 4A). SP also inhibited the phosphorylation of IκBα and ERK1/2 induced by ODN107 in a dose-dependent manner (Figure 4B). The results demonstrated that the inhibitory effect of SP on CpG ODN-induced cytokines release and NF-κB activation was associated with inhibition of phosphorylation of IκBα and ERK1/2.

### 2.4. SP Inhibits CpG ODN Internalization within RAW264.7 Cells

The internalization of CpG ODN is the first step in CpG ODN/TLR9-mediated cell activation, because TLR9 is an endosomal receptor [3,13]. Therefore, the influence of SP on ODN2006 internalization was further investigated. Two methods were used: laser confocal scanning observation and flow cytometry analysis.

**Figure 5:**
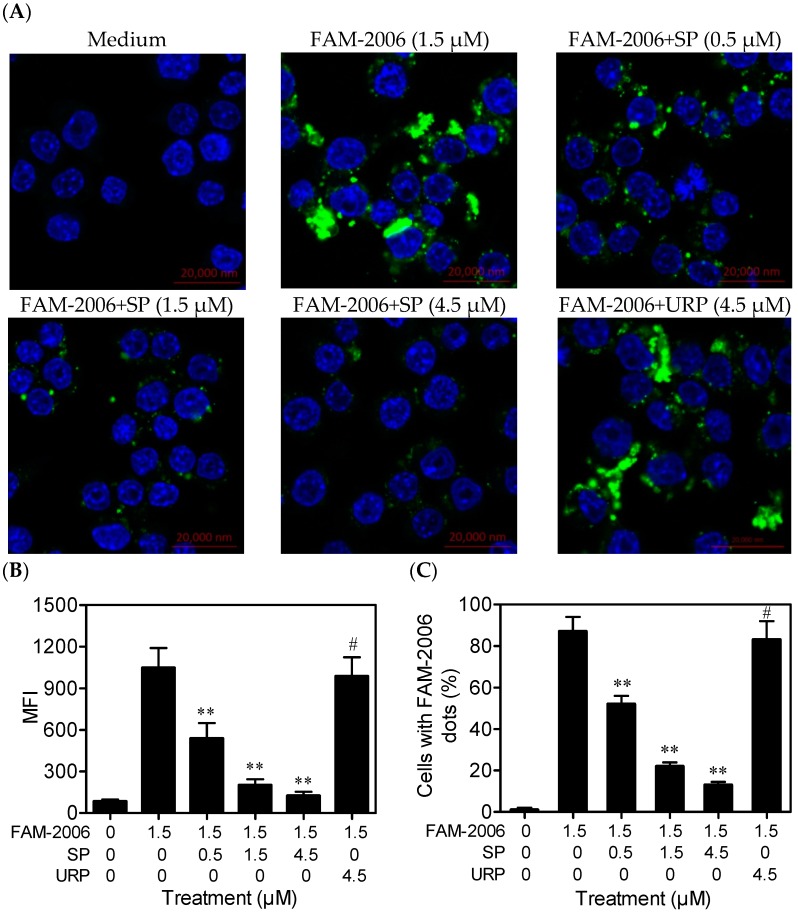
Synthetic peptide (SP) inhibited ODN2006 internalization, as assessed using laser confocal scanning (Scale bar = 20 μm). RAW264.7 cells (1 × 10^6^ cells) grown on 2-cm glass bottom dishes were treated with normal medium, 1.5 μM of FAM-labeled ODN2006 (FAM-2006) that had been preincubated with SP (0, 0.5, 1.5 or 4.5 μM) or unrelated peptide (URP; 4.5 μM) at 37 °C for 15 min. After treatment for 30 min, cells were washed with warm phosphate buffered saline (PBS) and fixed with 4% (*m*/*v*) paraformaldehyde, then stained with DAPI (4′,6-diamidino-2-phenylindole) (blue). The intracellular FAM-2006 (green) was observed using a laser confocal microscope (**A**); Mean fluorescence intensity (MFI) values (**B**) and the percentages of cells with FAM-2006 dots (green fluorescence) (**C**) were calculated by the ZENlite 2012 software (*n* = 100). “**”, *p* < 0.01; “#”, *p* > 0.05 *vs.* FAM-2006 alone.

**Figure 6:**
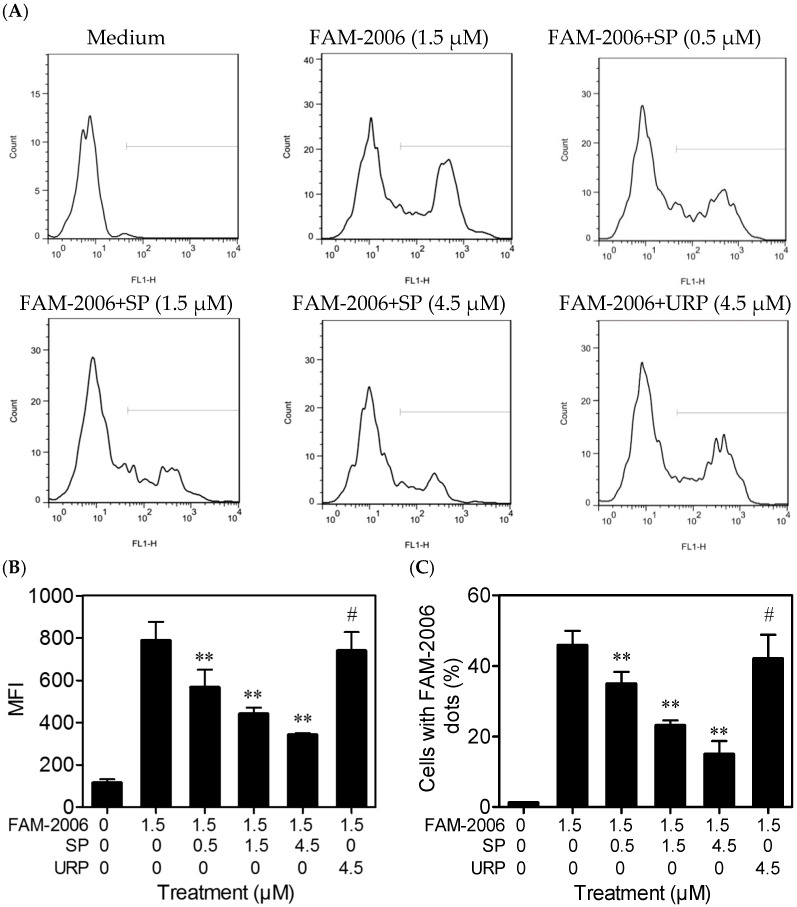
Synthetic peptide (SP) inhibited ODN2006 internalization, as assessed using flow cytometry analysis. RAW264.7 cells (5.0 × 10^5^ cells) grown in 12-well plates were treated as described in Figure 5. After 30 min, cells were washed with cold phosphate buffered saline (PBS), detached with trypsin/ethylenediaminetetraacetic acid (EDTA), counted, and immediately detected by flow cytometry (**A**). Mean fluorescence intensity (MFI) values (**B**) and the percentages of cells with FAM-2006 dots (green fluorescence) (**C**) were calculated using the Flowjo software (*n* = 3). “Count”, counted cells. “FL1-H”, the height of FL1 channel detecting FAM. “**”, *p* < 0.01; “#”, *p* > 0.05 *vs.* FAM-2006 alone.

The laser confocal scanning detected the intracellular fluorescence (green) produced by fluorescein amidite-labeled ODN2006 (FAM-2006). Untreated cells (Medium) had no green fluorescence, and almost all of the cells treated with FAM-2006 alone showed strong fluorescence. However, SP (0.5 μM) decreased the green fluorescence intensity within the cells (Figure 5A). Semiquantitative analysis results showed that SP decreased the mean fluorescence intensity (MFI) produced by FAM-2006 within the cells dose-dependently, whereas URP (4.5 μM) had no such effect (Figure 5B,C). Similar and coherent results were observed using flow cytometry (Figure 6), which further demonstrated that the inhibitory effect of SP on CpG ODN-induced cytokine release and NF-κB activation was related to its inhibition of CpG ODN internalization.

**Figure 7:**
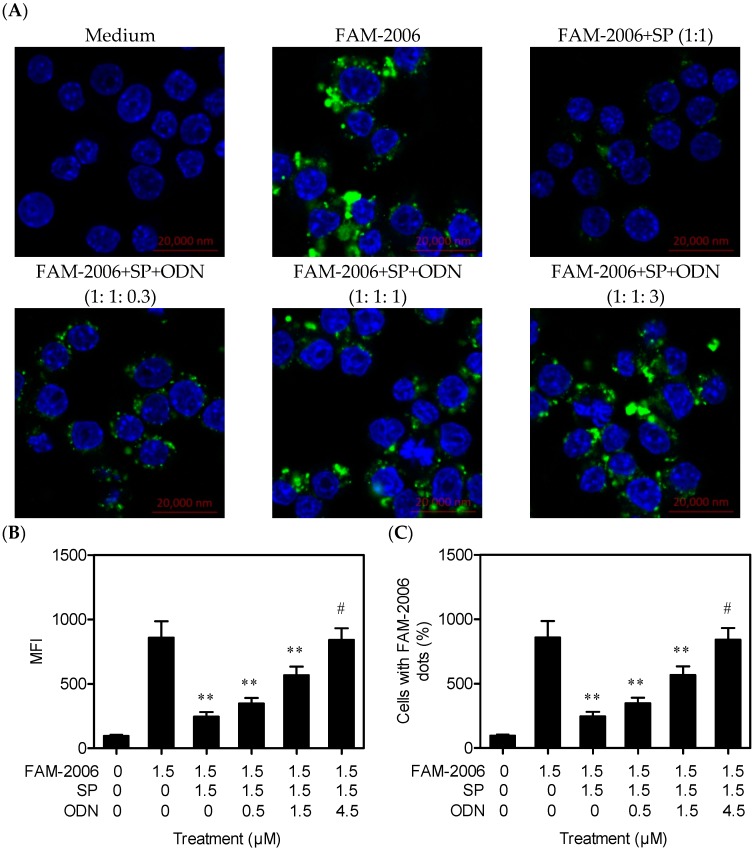
Non-labeled ODN2006 restored internalization of FAM-labeled ODN2006 preincubated with synthetic peptide (SP) (Scale bar = 20 μm). RAW264.7 cells (1.0 × 10^6^ cells) grown on 2-cm glass bottom dishes were treated with FAM-labeled ODN2006 (FAM-2006) (1.5 μM) that had been pre-incubated with SP (1.5 μM) and non-labeled ODN2006 (ODN; 0, 0.5, 1.5 or 4.5 μM) at 37 °C for 15 min. After another 30-minute incubation, cells were treated as described in Figure 5, then stained with DAPI (4′,6-diamidino-2-phenylindole) (blue). The intracellular FAM-2006 (green) (**A**), mean fluorescence intensity (MFI) values (**B**) and the percentages of cells with FAM-2006 dots (green fluorescence) (**C**) were observed as described in Figure 5 (*n* = 100). “**”, *p* < 0.01; “#”, *p* > 0.05 *vs.* FAM-2006 alone.

The above data from confocal microscopy and flow cytometry suggested that SP decreased ODN2006 internalization. However, the results could not exclude the possibility that SP might act on FAM not ODN2006 itself. Therefore, a competitive internalization experiment using non-labeled ODN2006 was performed. Non-labeled ODN2006 and FAM-2006 have same ability to bind SP, but FAM had no such ability. If SP specifically acted on ODN2006, non-labeled ODN2006 might increase FAM-2006 internalization, and the intracellular fluorescence intensity would increase. The results showed that non-labeled ODN2006 increased the MFI and the percentage of cells with green fluorescence in a dose-dependent manner. Importantly, 4.5 μM of non-labeled ODN2006 could completely restore cell internalization of FAM-2006 (1.5 μM) (Figure 7), suggesting that non-labeled ODN2006 competitively and completely bound SP. Therefore, FAM-2006 could completely enter the cells, resulting in a subsequent increase in intracellular fluorescence. These results clearly demonstrate that SP could specifically bind CpG ODN, leading to reduced CpG ODN internalization and inhibition of CpG ODN/TLR9-mediated signaling pathways.

### 2.5. SP Binds CpG ODN with High Affinity

**Figure 8:**
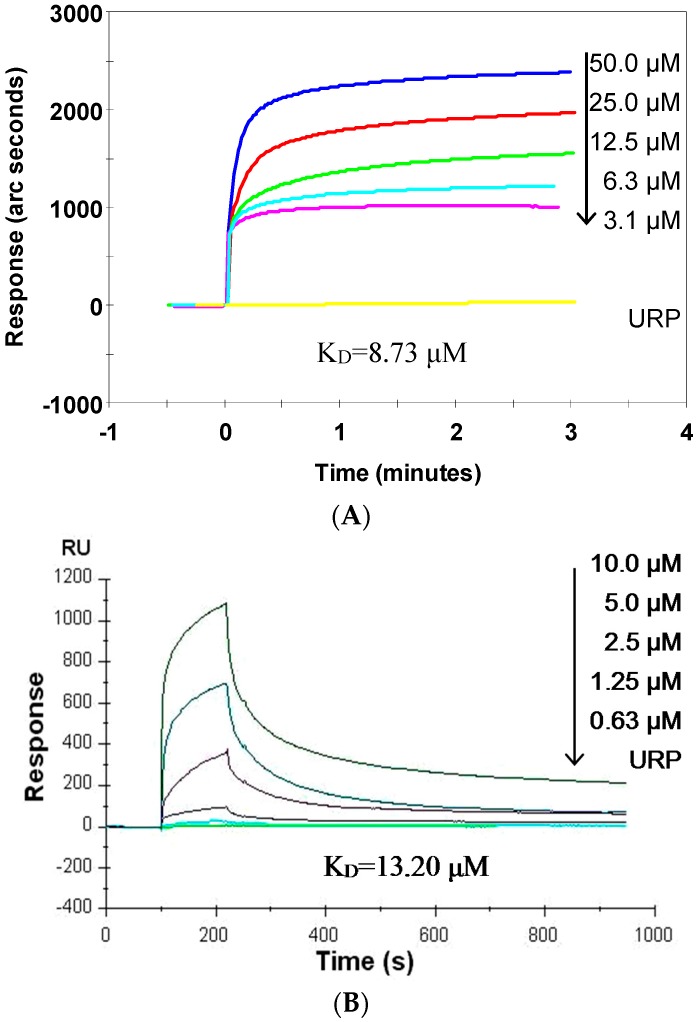
Synthetic peptide (SP) specifically binds ODN2006. (**A**) Binding curves generated by the IAsys biosensor. Biotinylated ODN2006 was immobilized on the surface of a streptavidin-coated cuvette. A series of concentration of SP (3.1, 6.3, 12.5, 25 or 50 μM) or unrelated peptide (URP) (50 μM) were added separately into the cuvette to generate the binding curves. Data were analyzed using FASTplot, and the dissociation equilibrium constant (K_D_) of SP was calculated using FASTfit; (**B**) Binding curves generated by the Biacore biosensor. Biotinylated ODN2006 was immobilized on a streptavidin-coated chip, and then a series of concentrations of SP (0.63, 1.25, 2.5, 5 or 10 μM) or URP (10 μM) were loaded separately to generate the binding curves. Data analysis and K_D_ calculation were performed using the BIAevaluation software.

The dissociation equilibrium constant (K_D_) value is considered as the gold standard to evaluate molecular interactions [3]. SP acts directly on ODN2006; therefore, the interaction between SP and ODN2006 was determined by two affinity experiments using an IAsys biosensor and a Biacore biosensor. The results from the IAsys biosensor experiments showed that SP had a strong binding capacity for ODN2006 (Figure 8A), and could bind ODN2006 in a dose-dependent manner. The K_D_ value of SP for ODN2006 was 8.73 μM. By contrast, URP showed no such response, indicating that SP specifically binds to ODN2006.

The results from the Biacore biosensor experiments also show that SP dose-dependently bound ODN2006 (Figure 8B). The K_D_ value was 13.2 μM, very close to that from the IAsys biosensor experiments. Therefore, the results of the two independent experiments demonstrated that SP could specifically bind ODN2006 with high affinity, further demonstrating that SP inhibited CpG ODN/TLR9-mediated pathways by decreasing CpG ODN internalization via binding CpG ODN directly.

### 2.6. Discussion

In our study, SP, a synthetic peptide corresponding to the LRR11 region of the TLR9 extracellular domain, was identified as a potential inhibitor of TLR9 signaling within macrophages. Herein, SP could significantly inhibit CpG ODN-induced release of proinflammatory cytokines (TNF-α and IL-6), activation of NF-κB, and phosphorylation of key upstream kinases (IκBα and ERK1/2) via decreasing CpG ODN internalization by directly binding CpG ODN.

CpG ODNs, which are bacterial genome-derived PAMPs recognized by endosomal TLR9, are closely associated with infectious diseases and autoimmune disorders [1,4]. Strategies to block the CpG ODN/TLR9 pathway have been demonstrated as potential treatments for infectious diseases, lupus nephritis and rheumatoid arthritis [9,14,15]. However, applications of known inhibitors, such as adalimumab [7], have been limited by their low-specificity, reflecting the fact that the signal molecules acted on by these inhibitors are also employed by pathways mediated by other TLRs [1]. Hence, more specific inhibitors should be investigated. The synthetic peptide from the ligand-binding domain of TLR9 is specific, and should be investigated as a candidate antagonist of CpG ODN.

TLR9 is a large transmembrane receptor. As a type I integral membrane glycoprotein, TLR9 consists of a pathogen-binding ECD and cytoplasmic Toll/interleukin-1 receptor domain, joined by a single transmembrane helix [16]. There are 25 LRRs in the ECD of hTLR9, five (LRR2, 5, 8, 11 and 20) with inserted sequences were predicted to bind CpG ODN [17]. Our previous results demonstrated that LRR11 is a crucial LRR for CpG ODN recognition [3]. Data from our and other groups showed that LRR 2, 5 and 8 also participate in the recognition of CpG ODN [3,10]. All the LRRs contribute to form the horseshoe shape that stabilizes the conformation of TLR9 and is critical for ligand binding [17]. Hence, the LRR11-peptide was proposed as a potential antagonist of CpG ODN because of its binding capacity for CpG ODN. Its influence on CpG ODN-induced activation of TLR9 signaling was further investigated by a series of cellular tests to evaluate proinflammatory cytokine release, NF-κB activation, kinases phosphorylation, and CpG ODN internalization. Note that SP was preincubated with CpG ODN to allow them to tightly bind together before adding the SP-CpG ODN mixture to RAW264.7 cells.

CpG ODN-induced diseases, such as sepsis and some autoimmune disorders, are triggered by uncontrolled release of proinflammatory cytokines, e.g., TNF-α and IL-6 [18]. Hence, inhibition of cytokine release is considered a good marker of blocked TLR9 signaling. Our previous reports indicated that both chloroquine and kukoamine B were inhibitors of CpG ODN-induced cytokine release, although they also showed similar inhibitory effects on LPS-induced cytokine release [19,20]. Herein, we firstly evaluated the inhibitory effect of SP on CpG ODN-induced cytokine release in the cellular tests. Two CpG ODNs: ODN2006 (a well-known stimulatory CpG ODN) and ODN107 (a stimulatory CpG ODN developed in our laboratory) were employed [21]. The results demonstrated that SP inhibited the release of TNF-α and IL-6 induced by these two CpG ODNs. However, SP is a selective inhibitor of CpG ODN-induced cytokine release because it had no influence on the cytokine release induced by LPS and PAM3. In addition, TNF-α and IL-6 release was only significantly inhibited by 1.5 and 4.5 μM of SP, suggesting the potency of SP on inhibiting cytokine release is dose-dependent. We used URP as a negative control, which is a part of LRR14 from TLR5 and contains several leucine residues. However, URP contains fewer positively charged residues (Arg-7) than SP (Arg-3, Lys-4, Arg-13 and Lys-14), which are critical for ligand binding, according to previous reports [3,10].

NF-κB is one of the most important inflammatory switches that comprise a series of transcription factors that regulate the expressions of various proinflammatory cytokines (IL-1, IL-6, IL-8 and TNF-α) [18]. NF-κB activation is depended on the degradation and phosphorylation of IκBα [12]. Our previous study showed that the inhibitors of IκBα phosphorylation and NF-κB activation, such as chloroquine and kukoamine B, also inhibited proinflammatory cytokines [19,20]. A previous report indicated desoxyrhapontigenin inhibited LPS-induced cytokine release by decreasing both of IκBα and ERK1/2 phosphorylation [22]. Herein, SP showed a significant inhibitory effect on CpG ODN-induced activation of NF-κB p65, which is considered the most crucial subunit of NF-κB [23]. Consistently, SP also decreased ODN2006-induced degradation and phosphorylation of IκBα, and phosphorylation of ERK1/2.

CpG ODN must be internalized within cells before activating TLR9 signaling and inducing cytokines release [20]. Thus, interfering with CpG ODN internalization probably leads to reduced activation of TLR9 signaling. A previous report indicated that inhibitors of clathrin-mediated phagocytosis, such as monodansylcadaverine and dynasore, had significant inhibitory effects on TLR signaling [24]. However, they had no potential for clinical use because of their distinct cell toxicity. Using confocal imaging and flow cytometry, SP was observed to decrease the fluorescence of FAM-2006 within RAW264.7 cells dose-dependently, suggesting that SP interfered with CpG ODN internalization. A competitive internalization experiment using non-labeled ODN to challenge FAM-2006 demonstrated that non-labeled ODN increased FAM-2006 internalization, suggesting that SP reduced CpG ODN internalization by directly binding to the CpG ODN not FAM. The above data clearly demonstrated that SP attenuated CpG ODN-induced TLR9 signaling by interfering with CpG ODN internalization within RAW264.7 cells. Considering that CpG ODN internalization is the first step of CpG ODN-mediated cell activation, the peptide’s effect lies upstream of CpG ODN/TLR9-mediated signaling.

The K_D_ value is the gold standard for molecular interactions. The smaller the K_D_ value, the higher the affinity between two molecules [20]. Herein, two widely used biosensor methods, IAsys Plus affinity biosensor and Biacore3000 system were used [3,25] to determine the K_D_ value of SP’s binding to a CpG ODN. The K_D_ values of SP for ODN2006 were 8.73 μM detected by IAsys method and 13.2 μM produced by Biacore3000 method, respectively. The K_D_ values generated by the two independent methods were similar. These data indicated that SP specifically bound ODN2006 and finally confirmed the previous prediction that SP indeed acts on CpG ODN. 

A previous report indicated that endosome acidification was required for TLR9 activation by CpG ODN [26]. However, whole TLR9 was reported to bind CpG ODN extracellularly in a non-acidic environment too [27,28]. Consistently, our results indicated that TLR9-LRR11 could bind CpG ODN in a neutral environment. Based on the observation that the inhibitory effect of SP on TLR9 signaling is dependent on preincubation of SP with CpG ODN, which interferes with CpG ODN internalization, we hypothesized that SP exerts its effect upstream of TLR9 signaling. Moreover, our previous report indicated that TLR9 bound to CpG ODN by the positively charged residues of LRR11, Arg-337 and Lys-338, through hydrogen bonding [3]. Accordingly, the binding capacity of SP with CpG ODN is probably associated with Arg-3 and Lys-4, the two positively charged residues in this peptide.

Known inhibitors of CpG ODN, such as chloroquine, and inhibitory CpG ODN are intracellular inhibitors interfering with the recognition of CpG ODN by TLR9 in the endosome, and their low-specificity probably reflects their influence on signaling through other TLRs [4,29]. However, our results demonstrated that SP is a selective inhibitor of CpG ODN. It could be used as a potential extracellular CpG ODN antagonist to block TLR9 signaling via binding CpG ODN, leading to reduced CpG ODN internalization and subsequently, lower signal activation. Additionally, we also provided a candidate method to screen for inhibitors of various PAMPs, which might play a significant role in the treatment of inflammatory and infectious diseases.

## 3. Experimental Section

### 3.1. Materials

Normal CpG ODN 2006 (abbreviated as ODN2006, 5′-TCGTCGTTTTGTCGTTTTGTCGTT-3′), 5′-biotin-labeled CpG ODN 2006 (abbreviated as biotin-ODN), FAM-labeled CpG ODN 2006 (abbreviated as FAM-2006) and CpG ODN107 (abbreviated as ODN107, 5′-TGGCGCGGGCGG-3′) with a nuclease-resistant phosphorothioate backbone, were synthesized by Invitrogen Ltd (Shanghai, China). The synthetic TLR9-LRR11 SP (QLRKLNLSFNYQKRVSFAHLSLAPSFGSLV) and URP (LQTLDLRDNALTTIHFIPSIPD) were synthesized by SBS Gene Technology (Beijing, China). Endotoxin-free DMEM (high glucose) medium and fetal calf serum (FCS) were supplied by GIBCO (Grand Island, NY, USA). Clarity Western ECL reagent was purchased from Bio-Rad Laboratories (Hercules, CA, USA). Primary and secondary antibodies were supplied by Cell Signaling Technology (Danvers, MA, USA). Mouse TNF-α and IL-6 ELISA Ready-SET-Go kits were obtained from eBioscience (San Diego, CA, USA). Trans^AM^ NF-κB ELISA kits for the p65 subunit were supplied by Active Motif (Carlsbad, CA, USA).

### 3.2. Cell Culture

The RAW264.7 cell line was purchased from American Type Culture Collection (ATCC, Manassas, VA, USA). Cells were cultured in DMEM (high glucose) medium containing 10% FCS in a 37 °C humidified atmosphere with 5% CO_2_. Cells diluted with 0.4% trypan blue in phosphate-buffered saline (PBS, 0.1 mM, pH 7.2–7.4) were counted using a hemocytometer.

### 3.3. Cytokines Release Assays

RAW264.7 cells (4.0 × 10^5^ cells) were incubated in 48-well plates for 4 h, and supernatants were replaced with 0.4 mL of serum-free DMEM medium containing 1.5 μM of ODN2006 or ODN107, which had been preincubated with 0, 0.5, 1.5 or 4.5 μM of SP or 4.5 μM of URP at 37 °C for 15 min. After incubation for 6 h, the supernatants were collected to detect TNF-α and IL-6 concentrations using corresponding ELISA kits (eBioscience).

### 3.4. NF-κB Activity Assay

RAW264.7 cells (5.0 × 10^6^ cells) were placed in six-well plates for 4 h, and the supernatants were then replaced with fresh medium containing 1.5 μM of ODN2006 or ODN107 that had been preincubated with SP (0, 0.5, 1.5, or 4.5 μM) or URP (4.5 μM) at 37 °C for 15 min. After incubation for 4 h, the cells were collected to extract nuclear proteins. Equal amounts of each sample were used to detect the DNA binding activity of the NF-κB p65 subunit using Trans^AM^ NF-κB ELISA kits (Active Motif), according to the manufacturer’s instructions.

### 3.5. Western Blotting

RAW264.7cells (1.0 × 10^7^ cells) were incubated in dishes for 4 h, and the supernatants were replaced with fresh medium containing 1.5 μM of ODN2006 that had been preincubated with 0.5, 1.5 or 4.5 μM of SP for 15 min. Cells were sequentially cultured for another 4 h and then harvested to extract total proteins. Equal amounts of proteins from each treatment were separated by SDS-PAGE, and transferred onto 0.22 μm PVDF membranes (Bio-Rad). The blots were blocked with 5% dry skim milk and then probed with anti-IκBα, anti-p-IκBα, anti-ERK, anti-p-ERK and anti-β-actin antibodies (Cell Signaling). The blots were subsequently incubated with goat anti-rabbit IgG antibody (Cell Signaling) and developed with Clarity Western ECL reagent (Bio-Rad) to detect chemiluminescence under a ChemiDoc™ Touch imaging system (Bio-Rad).

### 3.6. Laser Confocal Scanning

RAW264.7 cells (1.0 × 10^6^ cells) were grown on 2-cm glass bottom dishes for 4 h, and supernatants were replaced with 1 mL of serum-free DMEM medium containing FAM-2006 (1.5 μM) that had been pre-incubated with SP (0, 0.5, 1.5 or 4.5 μM) at 37 °C for 15 min. After treatment for 30 min, cells were washed with warm PBS three times, fixed with 4% (*m*/*v*) paraformaldehyde for 10 min, stained with 5 μg/mL of DAPI (4′,6-diamidino-2-phenylindole) for 5 min, and then washed with PBS three times. The intracellular FAM-2006 (green fluorescence) was observed using a laser confocal microscope. Mean fluorescence intensity (MFI) values were calculated by the ZEN lite 2012 software.

### 3.7. Flow Cytometry Analysis

RAW264.7 cells (5.0 × 10^5^ cells) were incubated in 12-well plates for 4 h, and then treated as described in the confocal scanning section. After treatment for 30 min, the cells were washed three times with cold PBS, detached with trypsin/Ethylenediaminetetraacetic acid (EDTA), counted, and then used immediately to detect CpG internalization by flow cytometry. MFI values and gate rates were calculated using the Flowjo software.

### 3.8. Binding Affinity Assays

The affinity assay was firstly carried out using an IAsys Plus affinity biosensor (Thermo Labsystem, Altrincham, Cheshire, UK), according to our previous report [3,20]. Biotinylated ODN2006 was immobilized on the surface of a streptavidin-coated cuvette according to the user manual. A series of concentrations of SP (3.1, 6.3, 12.5, 25, or 50 μM) or URP (50 μM) were added separately into the cuvette and allowed to bind for 3 min. The binding curve of each concentration of SP was generated. The cuvette was washed consecutively with 50 μL of PBS (0.01 M, pH 7.2) and 0.01 M HCl to regenerate the ODN2006-coated surface of cuvette. Binding curves were analyzed and visualized using FASTplot (Thermo), and the dissociation equilibrium constant (K_D_) of SP with ODN2006 was calculated using FASTfit (Thermo).

The Biacore3000 system (GE Healthcare, Uppsala, Sweden) was applied to reproduce the results of IAsys biosensor essentially as described previously [25]. In brief, biotinylated ODN2006 was diluted in running buffer (50 mM MES, 150 mM NaCl, 1 mM MgCl_2_ at pH 6.5) and loaded onto a streptavidin-coated chip. A series of concentrations of SP (0.63, 1.25, 2.5, 5.0 or 10 μM) or URP (10 μM) were diluted in 45 μL of running buffer, then loaded separately onto the chip at a flow rate of 10 μL/min. Binding was measured for 900 s (delay time, 100 s), and the chip was then regenerated using an NaOH-NaCl (50 mM and 1 M, respectively) solution and washed with running buffer. Data was analyzed and K_D_ value was calculated using BIAevaluation software (GE Healthcare).

### 3.9. Statistics

Cytokine concentrations and MFI values are shown as the means ± S.D. Student’s *t*-test was used for paired comparisons. Differences with *p* value less than 0.05 were considered statistically significant, and those less than 0.01 were considered highly statistically significant.

## 4. Conclusions

In conclusion, our results demonstrated that SP is a selective inhibitor of CpG ODN. It could be used as a potential extracellular CpG ODN antagonist to block TLR9 signaling via binding CpG ODN, leading to reduced CpG ODN internalization and, subsequently, less signal activation. Additionally, we also provided a method to screen inhibitors of various PAMPs, which might play a significant role in the treatment of inflammatory and infectious diseases.
